# Chlorhexidine alcohol base mouthrinse versus Chlorhexidine formaldehyde base mouthrinse efficacy on plaque control: Double blind, randomized clinical trials

**DOI:** 10.4317/medoral.17863

**Published:** 2012-12-10

**Authors:** Oumkeltoum Ennibi, Leila Lakhdar, Amal Bouziane, Yahia Bensouda, Redouane Abouqal

**Affiliations:** 1 Professor of Periodontology, Faculty of Dental Medicine, Mohammed V Souissi University, Rabat Morocco; 2 Assistant Professor, Faculty of Dental Medicine, Mohamed V Souissi University, Rabat Morocco; 3 Professor of Galenic pharmacology, Medical School, Mohammed V Souissi University, Rabat, Morocco; 4 Professor of Biostatistics and Clinical Research, Medical School, Mohammed V Souissi University, Rabat Morocco

## Abstract

Background: Chlorhexidine is well known for its antiplaque effect. However, the mouthrinse based chlorhexidine antiplaque efficiency may vary according to the formulation of the final product. The aim of the present study was to compare anti-plaque effectiveness of two commercial mouthrinses: 0.12 % Chlorhexidine alcohol base (CLX-A) versus a diluted 0.1% Chlorhexidine non-alcohol base with 0.1% of Formaldehyde (CLX-F). Material and Methods: the study was a seven day randomized, double-blind, placebo-controlled trial including 30 volunteers. At the start, all participants received a dental prophylaxis. Over 7 days experimental non-brushing period, during which subjects abstained from all forms of mechanical oral hygiene, one group test rinsed twice daily with 15ml of an alcohol base 0.12% Chlorhexidine mouthrinse. The second group test used 15ml of alcohol free 0.1% Chlorhexidine mouthrinse base 0.1% formaldehyde twice daily. The negative control group used a placebo. Plaque indexes were recorded in all volunteers prior to treatment at Day 0, 1 and 7. Results: After 7 days, the mean plaque index for the first group was 0.76±0.38 compared with a mean plaque index of 1.43±0.56 for the second group. The difference in plaque scores between the groups was statistically significant. Conclusion: the results of this study showed that rinsing with an alcohol base 0.12% Chlorhexidine mouthrinse is significantly different from rinsing with an alcohol free 0.1% Chlorhexidine mouthrinse on plaque inhibition.

** Key words:**Chlorhexidine, dental plaque, mouthrinse, alcohol, formaldehyde.

## Introduction

Since 1965, dental plaque has been considered to be the initiating factor in the development of gingivitis (Loë et al, 1965) (1). Therefore, plaque control has been the keystone of periodontal diseases’ prevention and treatment. Plaque control is based on using toothbrush (manual or electric); interdental brushes, floss, and toothpicks. These various tools are essential for oral hygiene and require a degree of motivation and skill for an effective and sustained use. In the absence of an adequate oral hygiene by mechanical devices, chemical products would act as a complementary or in some cases as an alternative method (Brecx 1997) (2).

Among antiseptics possessing an anti plaque effect, chlorhexidine is certainly the most tested one during the last decades. As a result of previous clinical studies, 0.2 % chlorhexidine mouthrinse (CLX) has become the international standard (3-8).

However, because of some side effects associated with this agent (tooth staining, taste disturbance), mouthrinses with a low level of this agent were proposed. Indeed, many studies have demonstrated that 0.1 % and 0.12 % CLX mouthrinse have a significant comparable effect on plaque reduction as 0.2 % CLX formulation (9-12).

These results showed the importance of the dose effect independently of the concentration. Therefore, it would seem desirable to use a CLX mouthrinse with low concentration but released in sufficient dose to preserve its action on plaque inhibition. Despite the concentration and the dose, the formulation of CLX mouthrinses can also influence plaque inhibition. Traditionally, CLX mouthrinses include alcohol. But, because of adverse effects of this agent, the alcohol was removed in some brands.

The aim of the present study was to investigate the effect on plaque inhibiting of two CLX mouthrinses: 0.12 % CLX alcohol base versus a diluted 0.1 % CLX non-alcohol base containing 0.1% of formaldehyde, and a placebo solution.

## Material and Methods

-Participants

To detect a minimum effect size of 0.6 (large effect) in mean plaque index variation at day 7, with 80% power and a two-sided 5% significance level, would require 30 patients (10 in each group).

The study included 30 volunteers among healthy dental students from the school of dentistry in Rabat, Morocco. Students were of both gender and aged 20 to 25 years. They were excluded from the study if any of the following were present: (1) less than 20 teeth; (2) pre-sence of periodontal disease (3) presence of factors of plaque retention (clinically unacceptable restorations, important carious, dental overlapping, removable prosthesis, faulty fixed prosthesis, orthodontic appliances), (4) associated systemic diseases (diabetes, heart disorders, blood diseases, VIH infection), (5) use of antibiotics or other anti-inflammatory drugs during the 3 latest months, (6) known allergy against components of mouth rinses, (7) pregnancy, (8) smoking.

The study was approved by the Ethical Committee of Biomedical Research of Mohamed 5 Souissi University, Rabat, Morocco (CERB) Ref: 1251/09.

All recruited students received complete information about the study and gave written informed consent before entering the study.

-Study design

This was a double-blind study with 3 parallel groups using 3 different solutions.

Volunteers were randomized to receive for 7 days either placebo or chlorhexidine mouthrinse alcohol base or Chlorhexidine mouthrinse formaldehyde base.

• The first test group used 15 ml of pure 0.12% CLX alcohol base (Group1: 0.12 % CLX-A).

• The second test group used 15 ml of diluted 0.1 % CLX non-alcohol base containing 0.1% of formaldehyde (Group2: 0.1 % CLX-F).

• The third control group used 15 ml of a placebo (Group3: P). The placebo is a solution containing 0.1g vanilla, 1g sorbitol, and water.

Mouthrinses were supplied in coded bottles containing 125 ml. The placebo was identically supplied.

This clinical trial was including two phases: pre-expe-rimental period of 14 days, and experimental period of 7 days.

-Pre-experimental period 

The aim of this phase was to obtain, on Day 0 (the day before the start of the experimental period), an oral cavity with low plaque deposits (PI< 1) and from gingival bleeding.

During this period, the volunteers were instructed to establish and maintain strict oral hygiene. Similar toothbrushes, toothpastes, and flosses were given to each one. They also received, during two weeks (Day -14 to Day 0), sessions of professional mechanical tooth cleaning to remove all visible plaque, calculus, and extrinsic tooth stain.

Plaque index (PI) was recorded at Day -14 and Day 0.

-Experimental period

A computer-regenerated randomisation list was drawn up by the statistician (RA). The dental practitioner (AB), responsible for seeing the participants, allocated the next available number on entry into the trial, and each participant collected his mouthrinse solution from the department of periodontology.

The clinical trial lasted 7 days (from Day 0 to Day 7). During this period all participants abstained from all mechanical oral hygiene procedures.

On Day 0, subjects were randomly distributed in three groups: two test groups and one negative control group, and were instructed to rinse twice daily with the allocated mouthrinse during the experimental period.

The clinical measurement: Plaque Index (PI) was recorded on Day 1 and Day 7.

On Day 7, all subjects were examined for the presence or absence of tooth staining, and were questioned about taste disturbances and mucosal sensitivities.

All measurements were carried out under the same conditions by the same qualified examiner (L.L), who was unaware of which mouthrinse was used by which participant. The participants were also blinded to the used mouthrinse.

The code was revealed to the periodontal practitioner once recruitment and data collection were completed.

-Statistical analysis

The quantitative variables (PI) were expressed on mean and standard deviation.

For the quantitative variables, the one way analysis of variance (ANOVA) was used for the comparison of the 3 groups followed by the Turkey test for the multiple comparisons between groups.

The qualitative variables (stains) were compared by the Fisher’s exact test.

The level of significance was based on p<0.05.

All statistical analyses were carried out using SPSS for windows 13.0 (SPSS, Chicago, IL).

## Results

All 30 subjects completed the 7 days rinse period (10 in the 0.12% CLX-A group, 10 in the 0.1% CLX-F group and 10 in the negative control group).

The mean plaque index (PI) on Day -14, Day 0, Day 1 and Day 7 is presented in  Table 1. There were no significant differences between the groups on Day -14, Day 0 and Day 1. But, on Day 7, the CLX-A group was significantly different from CLX-F group and the placebo group. Indeed, Scores of plaque overgrowth were less high with 0.12% CLX-A than with 0.1% CLX-F and placebo.

**Table 1:**
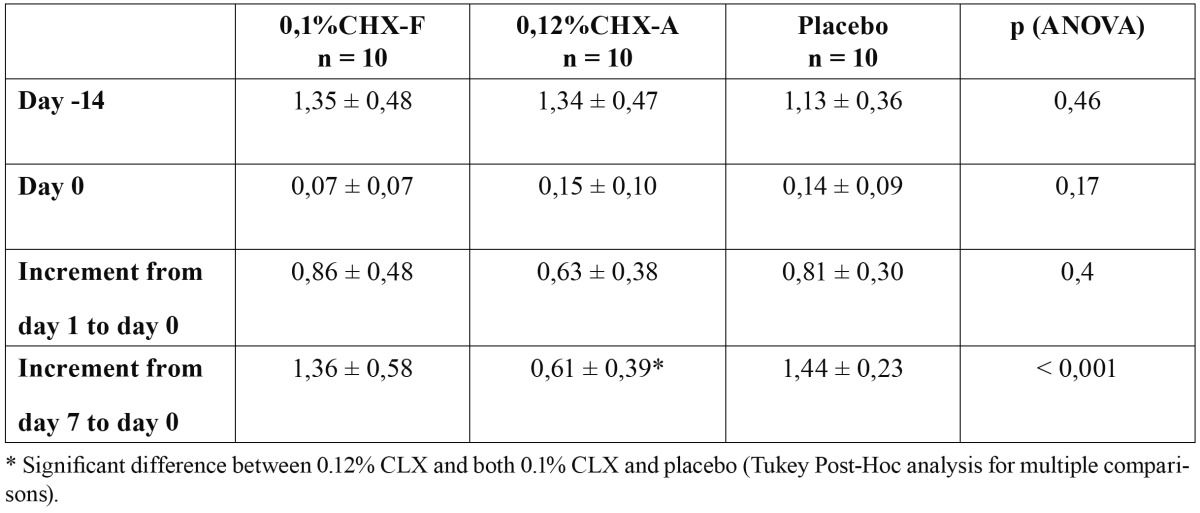
Mean plaque index variation (± standard deviation) during the experimental period.

The active solutions showed side effects in some subjects. Indeed, 2 participants (20%) complained about mouth burning and taste disturbance during and after rinsing with the 0.12% CLX solution (alcohol base). All side effects disappeared after stopping the mouthrinses and resuming daily oral hygiene procedures.

The tooth staining was recorded as present or absent. The scores evaluation did not show any significant difference among the tested product (Group 1 and Group 2) ( Table 2).

**Table 2:**
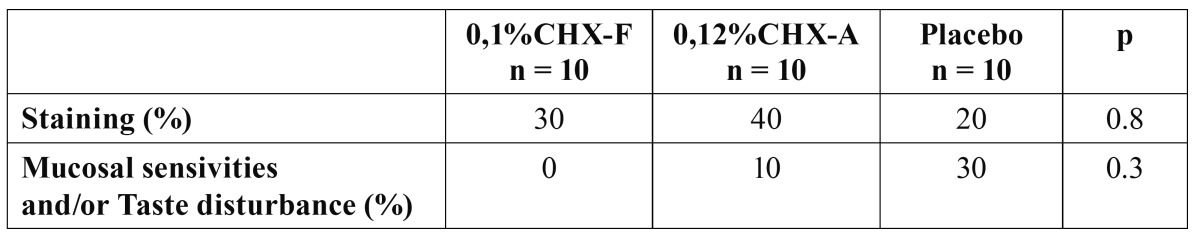
Side effects noticed in each group during the study.

## Discussion

Chlorhexidine mouthrinses have been the most common antiseptic agents used in periodontology for many years. Their efficiency as a suitable anti plaque agent has been proved by several authors both in “in vitro” and “in vivo” studies. Several regimens have required rinsing with 10 ml volumes of 0.2% CLX , for one minute, twice daily (4,11,13). However, this concentration induces tooth staining and taste aberration as local side effects (14). In order to reduce these side effects, different concentrations have been proposed. Thus, long-term clinical trials have shown no evident differences between 0.1%, 0.12% and 0.2% CHLX mouthrinses as anti-plaque and anti-gingivitis agents (15-19).

Our results indicate that, the 0.12% CLX-A mouthrinses resulted in considerably less plaque accumulation compared to diluted 0.1% CLX-F and the placebo. The same results were observed in a study of Addy et al. 1991 (20) which compared the effect of 0.12% and 0.1% CHX mouthrinses on plaque accumulation and found that mean scores for plaque were predominantly lower with the 0.12% mouthrinse than with 0.1%.

These controversies can be explained either by the dose prescribed or the formulation of the mouthrinse. Indeed, the dose of CLX seems to be important on plaque inhibition. Previous studies showed that the optimum dose of CLX is considered to be around 20mg twice daily; which balance the efficiency against local side effects and user acceptability (21-23). According to Keijser et al.,2003 (24), 0.12% CLX seems to be as effective as 0.2% if the volume of rinse was increased from 10 to 15 ml giving an 18mg dose on each occasion. Similar findings were reported by Smith et al 1995 (15) who showed that both 15 ml of 0.12% and 10 ml of 0.2% CLX have a significant efficiency against plaque accumulation compared to the control.

It should be also mentioned that in our study, the volunteers in group 2 rinsed with 15ml of 0.10 CLX-F, but had been asked to use a third of mouthrinse with two thirds of water according to manufactures’ instructions. This reduces the concentration of the used CLX-F to 0.03% CLX solution; which can be probably the reason for the poorer efficacy. Indeed, the plaque inhibitory efficacy of Chlorhexidine is dose related with a dose –response curve being significantly flat above doses of 5-6mg twice per day (Jenkins et al., 1994) (23).

Beside the concentration and the dose of Chlorhexidine mouthrinse, its formulation also influences the efficiency (25). Many chemical agents have been added to mouthrinse solutions like alcohol, Sodium fluoride, and Cetyl Peridium Chloride. Among these agents, alcohol is widely used as an antiseptic agent itself and as a dissolvent of other ingredients.

In this study 0.10% CLX-F (non alcohol) seems to be less efficient than the standard alcohol-containing chlorhexidine. This could be interpreted as CLX-A mouthrinses have slightly better anti plaque and anti gingivitis effects than CLX-F.

However, it should be remembered that CLX-non alcohol mouthrinses would favour continual plaque reduction and can be preferably used when there is intolerance to Alcohol. Indeed, many studies have shown an effectiveness of alcohol –free mouthrinse solutions in reducing plaque accumulation regarding to a placebo (16,26-27). In our study, the added component to the 0.1% CLX-non alcohol mouth rinse is Formaldehyde. This is an antiseptic agent widely used in endodontic product. However, its effect as an anti-plaque agent is not clear. Barnett et al. 2003 (28) had already pointed out, that the existence of a known substance (in this case, the chlorhexidine) does not guarantee activity and does not tell anything about its real action against plaque in vivo. This is mainly due to potential interactions-negative or positive ones- between the different components in a mouthrinse preparation.

Finally, in the present study, some side effects were reported by participants about taste perception and mouth burning during and after rinsing with the 0.12% CLX alcohol base solution. This is probably due to alcohol contained in the solution. Bolanowski et al. 1995 (29) found that an alcohol-containing CLX mouth rinse might even disturb the taste perception for many hours after rinsing (up to 4hours). Stains was also noticed in some subjects using either diluted 0.1% CLX-F or 0.12% CLX-A. This has been noted in the literature as an unwanted but common adverse event with the use of chlorhexidine mouth rinses. Lower dose or concentration does like standard 0.12% alcohol or non alcohol-containing CLX mouthrinse appears to provide little benefit to reduce dental staining (Francis et al., 1987) (30).

The use of the 0.1% CLX solution with formaldehyde (alcohol-free) showed, only in one subject, some local irritations: red patches on the back of the tongue, knowing that formaldehyde can prove to be irritating for tissues (31).

The double blind, randomized controlled design of this study showed significant results. However, the lack of crossover design is a limitation of the study.

## Conclusion

It can be concluded from this study that 0.12% CLX alcohol base mouthrinse is significantly more effective in inhibiting plaque than the diluted 0.1% CLX non-alcohol base containing formaldehyde mouthrinse.
